# Regulating mirroring of emotions: A social-specific mechanism?

**DOI:** 10.1177/17470218211049780

**Published:** 2021-10-08

**Authors:** Sophie Sowden, Divyush Khemka, Caroline Catmur

**Affiliations:** 1 School of Psychology, University of Birmingham, Birmingham, UK; 2 Department of Psychology, Institute of Psychiatry, Psychology & Neuroscience, King’s College London, London, UK

**Keywords:** Empathy for pain, personal distress, self-other control, emotional mirroring, empathic interference

## Abstract

There is evidence that humans mirror others’ emotional responses: brain responses to observed and experienced emotion overlap, and reaction time costs of observing others’ pain suggest that others’ emotional states interfere with our own. Such emotional mirroring requires regulation to prevent personal distress. However, currently it is unclear whether this “empathic interference effect” is uniquely social, arising only from the observation of human actors, or also from the observation of non-biological objects in “painful” states. Moreover, the degree to which this interference relates to individual differences in self-reported levels of empathy is yet to be revealed. We introduce a modified pain observation task, measuring empathic interference effects induced by observation of painful states applied to both biological and non-biological stimuli. An initial validation study (*N* = 50) confirmed that painful states applied to biological stimuli were rated explicitly as more painful than non-painful states applied to biological stimuli, and also than both painful and non-painful states applied to non-biological stimuli. Subsequently, across two independent discovery (*N* = 83) and replication (*N* = 80) samples, the task elicited slowing of response times during the observation of painful states when compared to non-painful states, but the magnitude of this effect did not differ between biological and non-biological stimuli. Little evidence was found for reliable relationships between empathic interference and self-reported empathy. Caution should therefore be taken in using the current task to pursue an individual differences approach to empathic interference, but the task shows promise for investigating the specificity of the mechanism involved in regulating emotional mirroring.

Humans are thought to “mirror” others’ emotional responses: much evidence for this claim comes from neuroimaging studies showing overlap in brain responses between observed and experienced emotion (Coll et al., 2017; Jackson et al., 2005; Singer et al., 2004). Overlap in observed and experienced emotional responses is also demonstrated by muscle responses during observation of pain to corresponding muscles in others (Avenanti et al., 2005). Furthermore, behavioural measures, whereby participants judge the laterality of an observed hand or foot in a pain or no-pain state, indicate a response time cost of observing others’ pain, even when performing an unrelated task, suggesting that others’ emotional states interfere with our own (Brewer et al., 2018; Gu et al., 2010, 2012). This is often referred to as an “empathic interference effect.”

Mirroring of others’ emotions may be adaptive, but when this tendency is not controlled, overt responding may occur, leading to personal distress—where individuals experience others’ negative emotions as their own (Avenanti et al., 2009). It has been argued, therefore, that successful social interaction, with feelings of sympathy as opposed to personal distress, may require regulation of the tendency to mirror the emotions of others (Decety & Lamm, 2009; Happé et al., 2017). Indeed, recent research has revealed that the ability to control competing representations of the self and others is important for social tasks including imitation-inhibition, perspective-taking, joint action, and lie detection (Boukarras et al., 2021; Brass et al., 2009; Sacheli et al., 2021; Santiesteban, Banissy, et al., 2012; Santiesteban et al., 2015; Santiesteban, White, et al., 2012; Sowden & Catmur, 2015; Sowden et al., 2015). For example, in the motor domain, it is important to regulate imitation of observed actions to prevent over-imitation and produce our own independent actions; necessary skills for successful social interaction. Recent data also suggest that the ability to regulate mirroring of others may be important in the emotional domain (de Guzman et al., 2016). de Guzman and colleagues demonstrated that training participants to increase self-other control in the motor domain led to a reduction in corticospinal responses indicative of personal distress when observing painful versus tactile stimulation to another’s hand.

Thus, regulation of the tendency to mirror others appears to play a role in successful social interaction. However, it is important to determine whether any such regulation mechanism is social-specific or instead whether it utilises general mechanisms of cognitive control. In the imitation domain, researchers have already attempted to isolate the ability to regulate action mirroring from more general cognitive control. For example, Catmur and Heyes (2011) developed a precisely matched non-social version of the imitation-inhibition task, whereby the ability to regulate spatially compatible responding can be dissociated from the ability to regulate imitation. A brain stimulation study using this task suggested that the regulation of action mirroring may be social-specific (Sowden & Catmur, 2015). In the emotion domain, however, task demands have not been well controlled to answer such questions of specificity. For example, longer response times to judge body part laterality when observing others’ pain (compared to a no-pain condition) could be due to the pain-inflicting object obscuring the body part, thus slowing laterality judgements. Furthermore, it is possible that the presence of interference effects in such laterality tasks may not be uniquely social at all, reflecting a more domain-general mechanism of cognitive control that operates regardless of the animacy of the target. Such a mechanism may be required to control interference in one’s laterality judgements whether observing biological or non-biological targets. A non-biological version of the pain observation task is therefore required to determine whether the regulation of responses to others’ pain utilises a mechanism that is specific to animate, biological stimuli.

The current study employed an adapted version of the laterality judgement pain observation task (Gu et al., 2010, 2012) incorporating closely matched non-biological trials to measure response times when observing pain to both biological and non-biological stimuli. Longer response times to identify the laterality of the task stimulus when observing pain than no-pain indicate empathic interference from the other’s pain. Thus, the ability to regulate such interference, and produce accurate laterality judgements, is indexed by a lesser response time cost in the pain compared to no-pain condition. Experiment 1 validated the new stimuli using explicit pain ratings, while Experiments 2 and 3 tested the pain observation task in both a discovery and independent replication sample. To determine whether empathic interference effects relate to individual differences in self-reported empathy, participants in Experiments 2 and 3 also completed a number of questionnaire measures.

The current study aims to measure the size of, and test the relationship between, empathic interference effects elicited by biological and non-biological objects, as well as to identify the specificity of any relationship between the ability to regulate uniquely social empathic interference and self-report measures of empathy. If a social-specific mechanism for empathic regulation does exist, we should expect to see greater empathic interference in response to the observation of pain to animate, biological stimuli than to non-biological stimuli. However, if interference effects are elicited by a more general cognitive control mechanism or simply by stimulus features such as a pain-inflicting object obscuring the view of the secondary object, we might expect to see equally prominent interference effects across both biological and non-biological stimuli.

A further crucial test of the independence of biological and non-biological interference effects will be whether the size of one effect predicts that of the other. If both types of interference effect are elicited by general cognitive control or attentional mechanisms, the non-biological interference effect should predict the size of the biological empathic interference effect, since both effects would be generated by the same regulatory process. If the two effects are not related in this way, it makes it more plausible that they are generated by different cognitive processes.

Complementary to the analysis above, under a hypothesis of a uniquely social form of emotion regulation in this task, we may also expect self-reported empathy to be associated with the biological interference effect, but not with that elicited by non-biological stimuli.

## Experiment 1

### Method

#### Participants

Fifty healthy adult participants, 10 male; mean ± standard deviation (*SD*) age = 25.5 ± 9.6 years, were recruited via the King’s College London research participation scheme and compensated with entry into a prize draw for a £50 Amazon gift voucher. Experimental procedures for all experiments were approved by the King’s College London Research Ethics Committee (HR-17/18-5296) and all participants gave informed consent prior to participation. We had no a priori knowledge of how painful the stimuli would be considered to be, nor of the likely variance in pain judgements. We therefore recruited a sample sufficiently large to provide 80% power to detect a medium effect size of d_z_ = 0.4 at an alpha level of .05.

#### Stimuli

**Figure 1.**
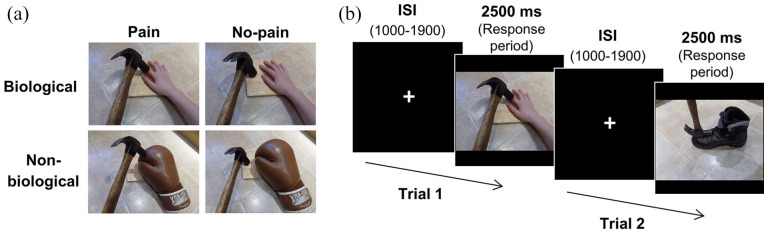
(a) Example stimuli for all four conditions of the pain observation task. (b) Trial structure for Experiments 2 and 3: Trial 1 represents a biological pain trial, while Trial 2 represents a non-biological “pain” trial. Interstimulus intervals (ISI) varied between 1000 and 1900 ms and responses were made during the stimulus presentation period.

New stimuli, based on the original task (Gu et al., 2010, 2012), were created to produce biological and non-biological variants of the pain observation task. During each trial (see Figure 1a), participants were presented with an image of a left or right hand, foot, glove or boot (target) depicted in either a “pain” or “no-pain” state. For example, a knife could be observed in contact with the target (pain state) or not in contact with the target (no-pain state). Hands and feet served as the biological targets with gloves and boots as the matched non-biological targets. Biological and non-biological targets were depicted in precisely the same “pain” and “no-pain” scenarios. Gloves and boots were used as our non-biological control stimuli as they most closely match the visual properties of hands and feet while also possessing the same feature of laterality (existing in left and right forms) which is crucial for responses required in the current task. While we recognise that no pain is inflicted in the case of the non-biological objects, we use the condition terms “pain” and “no-pain” for consistency across biological and non-biological conditions and in line with terms used in the literature. Forty stimuli were produced for each of the four conditions (biological pain; biological no-pain; non-biological “pain”; non-biological “no-pain”). Half the trials depicted a left and half a right target, equally distributed across the four conditions. Left and right target stimuli were a direct mirror of one another along the vertical axis. The targets were shown from a variety of perspectives; however, the perspective was always controlled across scenarios, such that the four images for each scenario (biological pain; biological no-pain; non-biological “pain”; non-biological “no-pain”) always showed the hand/glove/foot/boot from the same perspective, as illustrated in Figure 1a.

#### Procedure

Stimuli were presented and responses recorded via Qualtrics (Qualtrics, Provo, UT) and the experiment was completed online. Each participant was presented with a subset of 40 of the 160 task stimuli (with each participant rating an equal number of pain/no-pain/biological/non-biological stimuli), in a randomised order. On each trial, they were asked “Please rate how painful you find this image on a scale from 1 (not painful) to 10 (extremely painful).” These responses were made on a slider scale with anchors of 1 and 10 labelled at the ends of the scale. Each stimulus was presented for an unlimited time until response, with the response slider positioned below the image.

### Results

Mean painfulness ratings were calculated for each participant for each condition and are displayed in Table 1. These were subjected to repeated-measures ANOVA with factors of animacy (biological, non-biological) and pain (pain, no-pain). Main effects of both animacy (*F*_1,49_ = 207.23, *p* < .001, η_p_^2^ = .809), and pain (*F*_1,49_ = 124.88, *p* < .001, η_p_^2^ = .718), were observed, with biological stimuli producing higher painfulness ratings than non-biological stimuli, and pain trials higher ratings than no-pain trials. There was, however, a significant interaction between animacy and pain (*F*_1,49_ = 136.61, *p* < .001, η_p_^2^ = .736), whereby the effect of pain on the painfulness ratings was greater for biological than non-biological stimuli, although the simple effect of pain was present for both biological (*t*_49_ = 12.79, *p* < .001, *d_z_* = 1.81, Hedges’ *g_av_* = 1.87) and non-biological (*t*_49_ = 5.46, *p* < .001, *d_z_* = 0.77, Hedges’ *g_av_* = 0.72) stimuli. Importantly, the painfulness ratings for the biological pain stimuli were significantly higher than all other stimulus types (all *p* < .001), indicating that the biological pain stimuli were rated explicitly as more painful than stimuli from the other three conditions. Furthermore, the absolute painfulness ratings for the non-biological “pain” stimuli were low (mean 2.2 out of 10) and did not differ from those for the biological no-pain stimuli (*p* = .4).

**Table 1.** Mean ± standard error of the mean pain ratings for each stimulus type for Experiment 1, and response times (ms) and error rates (%) for each condition for Experiments 2 and 3.

	Biological		Non-biological	
	Pain	No-pain	“Pain”	“No-pain”
Experiment 1				
Pain ratings	5.3 ± 0.3	2.1 ± 0.2	2.2 ± 0.2	1.4 ± 0.1
Experiment 2				
Response times	873.9 ± 17.5	840.0 ± 16.9	859.4 ± 17.0	827.0 ± 16.0
Error rates	3.6 ± 0.5	4.0 ± 0.5	6.6 ± 0.6	5.8 ± 0.5
Experiment 3				
Response times	820.0 ± 18.9	788.6 ± 17.3	799.6 ± 16.2	772.2 ± 16.5
Error rates	4.8 ± 0.6	4.6 ± 0.6	8.3 ± 0.7	7.3 ± 0.6

Due to the non-continuous nature of the rating scale used in this study, non-parametric Wilcoxon Signed-Rank tests were also performed to verify the comparisons above. The simple effect of pain was present for both biological (*Z* = 6.15, *p* < .001) and non-biological (*Z* = 5.57, *p* < .001) stimuli, and the painfulness ratings for the biological pain stimuli were significantly higher than all other stimulus types (all *p* < .001); whereas the painfulness ratings for the non-biological “pain” stimuli did not differ from those for the biological no-pain stimuli (*Z* = 0.56, *p* = .58).

### Discussion

Explicit painfulness ratings of the stimuli in the four conditions indicated that biological pain stimuli were considered more painful than stimuli in all other conditions. Although there was an effect of painfulness on the non-biological stimuli, the effect size was medium-to-small, and the painfulness ratings for the non-biological “pain” stimuli did not differ from those for the biological no-pain stimuli, indicating that the non-biological “pain” stimuli were considered no more painful than the biological no-pain stimuli.

The rating scale asked participants to indicate how painful they found each image. However, it is possible that this wording could be interpreted as relating to either the model’s pain or the participant’s own pain. This should not have had a direct impact on the current results since each participant is likely to have interpreted the question in the same way for all four conditions, but future studies might choose to include two separate questions to index both aspects of the empathic emotional response.

The above point notwithstanding, these data suggest that the stimuli provide a valid manipulation of painfulness, with the biological pain stimuli being rated, as intended, as the most painful. In two studies using a similar paradigm (Gu & Han, 2007; Gu et al., 2010) pain ratings were around the mid-point of the scale (3.7 out of 6 and 3.5 out of 5 respectively). These are not dissimilar to our current data for the biological pain condition (5.3 out of 10). Furthermore, the interaction between pain and animacy indicates that the effect of pain is significantly greater for the biological stimuli, again as intended. Thus, when used in the pain observation task in Experiments 2 and 3, the biological stimuli are expected to produce an empathic interference effect (longer response times to identify the laterality of the stimulus when observing pain than when observing no-pain).

If the empathic interference effect is found to be specific to, or greater for, the biological stimuli, this will support the suggestion that this effect is governed specifically by the observation of pain delivered to biological stimuli. In contrast, if the empathic interference effect is equally prominent for the non-biological stimuli, then given that the explicit pain ratings did differ between biological and non-biological stimuli, such a result would suggest that features of the stimuli other than their painfulness are sufficient to produce an empathic interference effect.

## Experiment 2

### Method

#### Participants

Ninety-five adult participants were recruited via the King’s College London research participation scheme, were remunerated £6 for participation and all gave written informed consent prior to participation. Two participants did not complete the study. Participants were excluded if they made more than 25% errors on the task (*N* = 6), if their mean response time was more than 2.5 standard deviations (*SD*) from the group mean (*N* = 3), or if they were a multivariate outlier (Tabachnick & Fidell, 2001) on the crucial comparison between the biological and non-biological interference effects (*N* = 1). The final sample comprised 83 participants (19 male, 6 left-handed; mean ± *SD* age = 25.3 ± 7.7 years). A power calculation based on the smallest effect size (*d_z_* = 0.5) for the empathic interference effect reported by Brewer et al. (2018) indicated that a sample of 32 participants would provide 80% power at an alpha level of .05. However, this effect was measured for biological stimuli, and we had no a priori knowledge of whether the effect size for non-biological stimuli would be equally large. We therefore recruited a sample sufficiently large to provide 80% power to detect a small to medium effect size of d_z_ = 0.3 at an alpha level of .05, allowing us to detect an effect substantially smaller than that reported by Brewer et al.

#### Procedure

Participants completed the pain observation task, along with another computerised task unrelated to the current research question, in counterbalanced order. The computerised tasks were followed by a series of questionnaires assessing self-reported empathy including the Interpersonal Reactivity Index (IRI; Davis, 1980), the Empathy for Pain Scale (EPS; Giummarra et al., 2015) and the Questionnaire of Cognitive and Affective Empathy (QCAE; Reniers et al., 2009), along with two further questionnaires unrelated to the current research question. The testing session lasted approximately 45 min and took place in person in King’s College London Psychology testing cubicles.

The pain observation task was programmed using Cogent and Cogent Graphics (Wellcome Department of Imaging Neuroscience) for MATLAB (MathWorks, Massachusetts, USA) and stimuli were presented in colour on a black background, using a Dell Optiplex 9030 AIO with 23-inch monitor (Dell Inc, Texas, USA), running Windows 10. The participant was seated 80 cm from the computer monitor during task completion and responses were made using an external keyboard.

Each trial (see Figure 1b) began with the presentation of a fixation cross for a variable ISI (1,000–1,900 ms), followed by the presentation of the target stimulus for 2,500 ms. Participants were instructed to respond on each trial during the presentation of the target stimulus as quickly and accurately as possible, however, the full 2,500 ms presentation stimulus time elapsed before the start of a new trial. They responded regarding the laterality of the target, i.e., whether the image depicted a left or a right hand/foot/glove/boot, by pressing the left or right arrow keys with the index fingers of their left and right hands. Therefore, whether a biological or non-biological target was observed (animacy factor) in a pain or no-pain state (pain factor) was formally task-irrelevant, while the laterality of the target was task-relevant. Participants began by completing 10 practice trials, equally distributed across the pain/no-pain and biological/non-biological conditions, in random order. The main task comprised 160 trials completed in two blocks of approximately 5 min each. Forty trials were presented for each of the four conditions (biological pain; biological no-pain; non-biological “pain”; non-biological “no-pain”). Eighty trials depicted a left target and 80 trials a right target, equally distributed across the four conditions. Trials were equally distributed across blocks and presented in random order within blocks.

### Results

For each participant, error trials, and trials where response times were more than 2.5SD from the participant’s mean, were removed prior to calculation of mean response times for each condition. Response times and error rates are displayed in Table 1.

Response times were subjected to repeated-measures ANOVA with factors of animacy (biological, non-biological) and pain (pain, no-pain). Main effects of both animacy (*F*_1,82_ = 6.11, *p* = .015, η_p_^2^ = .069) and pain (*F*_1,82_ = 74.69, *p* < .001, η_p_^2^ = .477) were observed, with biological stimuli producing slower response times than non-biological stimuli, and pain trials producing slower response times than no-pain trials. The simple effect of pain was significant for both biological (*t*_82_ = 6.12, *p* < .001, *d_z_* = 0.67, Hedges’ *g_av_* = 0.21) and non-biological (*t*_82_ = 6.50, *p* < .001, *d_z_* = 0.70, Hedges’ *g_av_* = 0.21) stimuli. No interaction was observed.

Error rates were subjected to the same ANOVA. A main effect of animacy (*F*_1,82_ = 35.05, *p* < .001, η_p_^2^ = .299) was observed, with biological stimuli producing fewer errors than non-biological stimuli. Non-parametric comparisons confirmed this result (*Z* = 2.68, *p* = .007). No other effects reached significance.

The ability to regulate responses to others’ pain was calculated by subtracting no-pain from pain response times, yielding an “empathic interference effect,” for biological and non-biological stimuli. To determine the relationship between the biological and non-biological empathic interference effects, along with the relationship between these measures and the empathy measures, two regression models were constructed. The first had the biological empathic interference effect as the dependent variable. At the first level, the non-biological empathic interference effect was entered as a predictor, followed by the questionnaire measures (broken down into subscales) at the next level. The second model had the non-biological empathic interference effect as the dependent variable, the biological empathic interference effect as first level and questionnaire measures as second level predictors. See Table 2 for regression results and simple correlations.

**Table 2.** Regression results and simple correlations for regression models predicting the biological empathic interference effect (Columns 2–6) and the non-biological empathic interference effect (Columns 7–11) in Experiment 2.

Predictor	Dependent variable: biological empathic interference					Dependent variable: non-biological empathic interference				
	Regression			Simple correlation		Regression			Simple correlation	
	β	t	*p*	r	p	β	t	*p*	r	*p*
Non-biological empathic interference	.060	0.538	.592	.060	.296	—	—	—	—	—
Biological empathic interference	—	—	—	—	—	.060	0.538	.592	.060	.296
QCAE perspective taking	−.178	−1.284	.203	−.029	.397	.149	1.052	.296	.141	.101
QCAE online simulation	.094	0.526	.600	.072	258	−.012	−0.067	.946	.044	.347
QCAE emotion contagion	.315	2.017	.048*	.232	.017*	.232	1.450	.152	.057	.306
QCAE proximal responsivity	−.195	−1.032	.306	.135	.112	−.168	−0.877	.383	−.096	.193
QCAE peripheral responsivity	−.190	−1.184	.241	−.100	.185	−.354	−2.237	.029*	−.208	.029*
IRI fantasy	.174	1.016	.313	.009	.467	.239	1.39	.169	−.028	.402
IRI empathic concern	.336	2.031	.046*	.238	.015*	−.027	−0.157	.876	−.019	.431
IRI perspective taking	−.053	−0.306	.760	.057	.305	.084	0.475	.636	.013	.455
IRI personal distress	−.017	−0.116	.908	.106	.169	−.055	−0.378	.706	−.047	.336
EPS affective distress	−.045	−0.290	.772	.075	.250	−.014	−0.09	.929	−.013	.453
EPS empathic concern	.010	0.065	.949	.185	.047*	−.014	−0.087	.931	.037	.371
EPS vicarious pain	−.020	−0.136	.892	.063	.286	−.102	−0.685	.495	−.081	.233

QCAE: Questionnaire of Cognitive and Affective Empathy; IRI: Interpersonal Reactivity Index; EPS: Empathy for Pain Scale.

* Denotes significant at *p* < .05.

**Figure 2.**
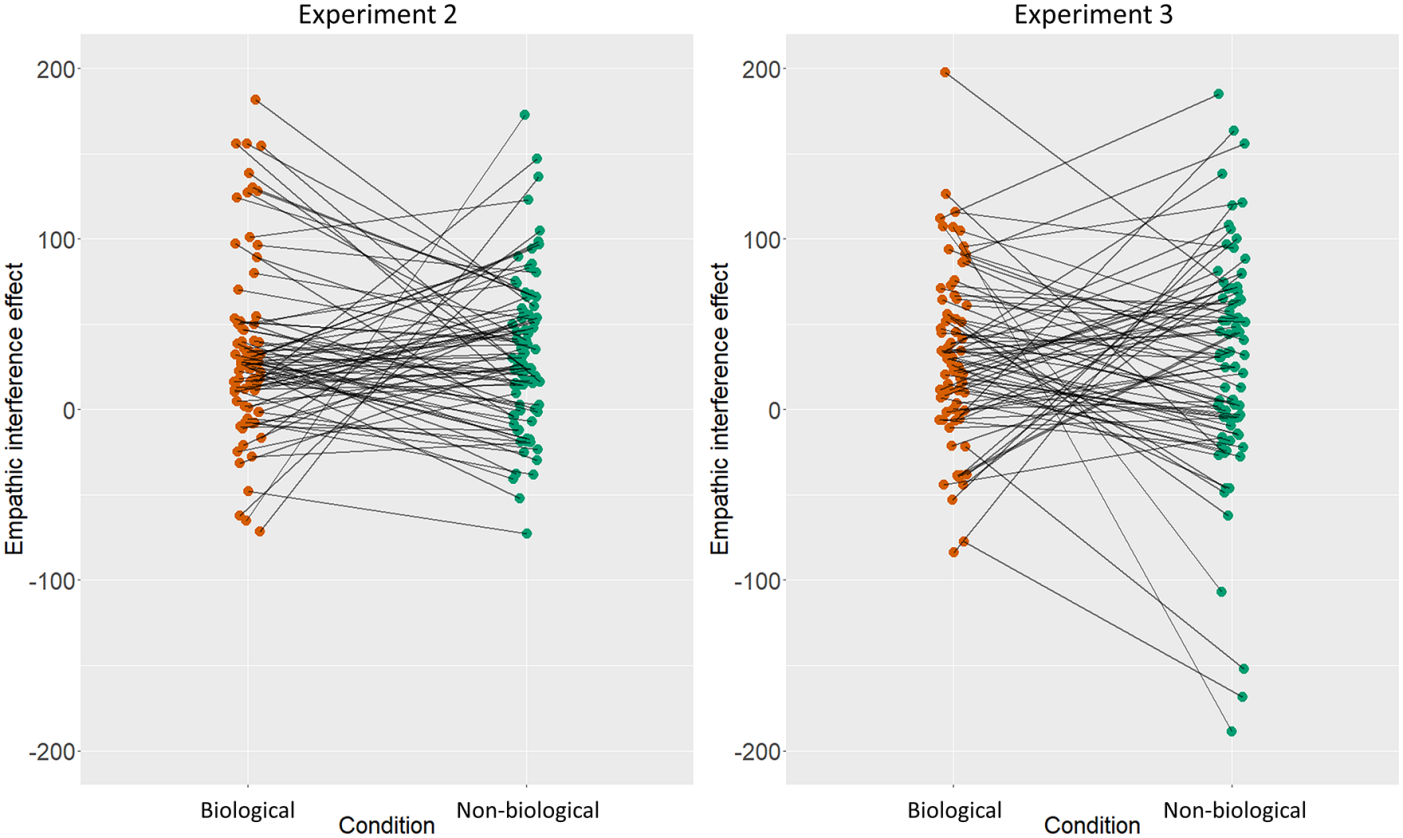
Experiment 2 (left) and Experiment 3 (right) individual participant level empathic interference effects for both biological and non-biological conditions.

As Table 2 indicates, the non-biological empathic interference effect was not a significant predictor of the biological empathic interference effect, with a Bayesian simple correlation revealing moderate evidence in favour of the null (BF_01_ = 6.33). (Bayes Factors [BF01] here provide a ratio of likelihood for the observed data under the null compared to the alternative hypothesis. Values of 3-10 are taken as moderate evidence for the null hypothesis; Lee & Wagenmakers, 2014). However, both the QCAE emotional contagion and the IRI empathic concern subscales were significant predictors once the non-biological interference effect was controlled for. The only significant predictor of the non-biological empathic interference effect was the QCAE peripheral responsivity subscale, which showed a negative relationship with the non-biological interference effect. See Figure 2 for individual participant level biological and non-biological interference effects.

### Discussion

Experiment 2 aimed to determine, first, whether the empathic interference effect that has been reported in previous pain observation studies (Brewer et al., 2018; Gu et al., 2010, 2012) is specific to animate, biological stimuli, and thus whether the ability to regulate the mirroring of emotions can be considered a social-specific mechanism or the result of a more general mechanism of cognitive control. Participants made speeded laterality judgements to biological and non-biological stimuli in painful and non-painful states. Despite explicit ratings of painfulness being higher for the biological pain stimuli than for all other conditions (Experiment 1), the empathic interference effect (longer response times when observing pain compared to no-pain stimuli) was of equal magnitude in the biological and the non-biological conditions. This result is consistent with the suggestion that the previously reported empathic interference effect, and thus the regulation of interference for biological stimuli in this task, does not necessarily index a social-specific process. That is, response time differences of equal magnitude were generated when observing biological and non-biological stimuli in “pain” versus “no-pain” states. This may indicate that empathic interference effects are the result of either stimulus features or a more domain-general cognitive control mechanism; or alternatively, that two different mechanisms generate and/or regulate interference effects of similar magnitude for biological and for non-biological stimuli.

The second aim of this experiment was to test these alternative accounts by examining the relationship between the ability to regulate empathic interference effects elicited by biological and non-biological stimuli. The regression analysis indicated that the magnitude of the empathic interference effect for biological stimuli was not predicted by that for non-biological stimuli. This result supports the second explanation above: that it is possible that the biological and non-biological interference effects may be regulated by two different cognitive processes.

The final aim of Experiment 2 was to test the specificity of any relationship between the ability to regulate biological empathic interference and self-report measures of empathy, when controlling for the ability to regulate non-biological interference. Three of the self-report subscales were associated with the biological empathic interference effect, and two of those associations (with the emotion contagion subscale of the QCAE and with the empathic concern subscale of the IRI) remained significant when controlling for non-biological empathic interference. In both cases, higher self-reported emotion contagion or empathic concern predicted a larger empathic interference effect, indicative of reduced regulation of emotional mirroring. The IRI empathic concern subscale contains items such as “I often have tender, concerned feelings for people less fortunate than me” whereas the QCAE emotion contagion subscale refers more directly to the regulation of emotional mirroring, containing items such as “People I am with have a strong influence on my mood.” The association between these subscales and the biological, but not the non-biological, empathic interference effect lends further validity to the suggestion that the biological interference effect measures social-specific empathic regulation.

Only one subscale was associated with the non-biological empathic interference effect. The QCAE peripheral responsivity subscale contains items such as “I often get deeply involved with the feelings of a character in a film, play, or novel” and “I usually stay emotionally detached when watching a film” (reverse coded). Participants who scored highly on this subscale showed smaller non-biological interference effects. This negative association is surprising as it suggests that individuals who do not easily detach themselves from external emotional events, may show better domain-general cognitive control. However, this finding requires replication.

In Experiment 3 we sought to replicate the findings of Experiment 2 in an independent sample. Due to time constraints, in this study only one self-report empathy measure could be administered. As the QCAE emotion contagion subscale seems more directly relevant to the regulation of emotional mirroring, and furthermore as the QCAE was the only questionnaire associated with the non-biological interference effect, we chose to administer the QCAE in Experiment 3.

## Experiment 3

### Method

The same method of recruitment as in Experiment 2 was used to recruit an independent sample of 86 healthy adult participants, with all providing written informed consent prior to participation. The effect sizes for both the biological and non-biological empathic interference effects in Experiment 2 were of similar magnitudes, with *d_z_* = 0.67 and 0.70 respectively. A sample of 18 participants would provide 80% power to detect an effect of d_z_ = 0.67 at an alpha level of .05. However, to improve the estimate of the effect size, we chose to replicate the sample size of Experiment 2 and recruited a sample sufficiently large to provide 80% power to detect a small to medium effect size of *d_z_* = 0.3 at an alpha level of .05. Exclusion criteria were applied as in Experiment 2 (*N* = 6). The final sample comprised 80 participants (23 male, 8 left-handed; mean ± *SD* age = 22.9 ± 5.9 years).

Participants completed the same pain observation task as in Experiment 2, alongside the second computerised task in counterbalanced order. They then completed a modified battery of questionnaires including the QCAE and four other scales unrelated to the current research question. The testing session again lasted approximately 45 min and took place in person in King’s College London Psychology testing cubicles.

### Results

For each participant, error trials, and trials where response times were more than 2.5SD from the participant’s mean, were removed prior to calculation of mean response times for each condition. Response times and error rates are displayed in Table 1.

Response times were subjected to repeated-measures ANOVA with factors of animacy (biological, non-biological) and pain (pain, no-pain). Main effects of both animacy (*F*_1,79_ = 15.36, *p* < .001, η_p_^2^ = .163) and pain (*F*_1,79_ = 39.87, *p* < .001, η_p_^2^ = .335) were observed, with biological stimuli producing slower response times than non-biological stimuli, and pain trials producing slower response times than no-pain trials. The simple effect of pain was significant for both biological (*t*_79_ = 5.78, *p* < .001, *d_z_* = 0.64, Hedges’ *g_av_* = 0.19) and non-biological (*t*_79_ = 3.71, *p* < .001, *d_z_* = 0.41, Hedges’ *g_av_* = 0.19) stimuli. No interaction was observed.

Error rates were subjected to the same ANOVA. A main effect of animacy (*F*_1,79_ = 50.53, *p* < .001, η_p_^2^ = .390) was observed, with biological stimuli producing fewer errors than non-biological stimuli. Non-parametric comparisons confirmed this result (*Z* = 5.69, *p* < .001). No other effects reached significance.

As in Experiment 2, the empathic interference effect was calculated by subtracting no-pain from pain laterality judgement response times for biological and non-biological stimuli. To determine the relationship between the ability to regulate biological and non-biological empathic interference and the questionnaire measures, two regression models were constructed in the same way as in Experiment 2 (see Table 3).

**Table 3.** Regression results and simple correlations for regression models predicting the biological empathic interference effect (Columns 2–6) and the non-biological empathic interference effect (Columns 7–11) in Experiment 3.

Predictor	Dependent variable: biological empathic interference					Dependent variable: non-biological empathic interference				
	Regression			Simple correlation		Regression			Simple correlation	
	β	T	*P*	r	p	β	t	*p*	r	p
Non-biological empathic interference	.033	0.294	.769	.033	.385	—	—	—	—	—
Biological empathic interference	—	—	—	—	—	.033	0.294	0.769	.033	.385
QCAE perspective taking	−.106	−0.825	.412	−.168	.068	.076	0.588	.558	.104	.179
QCAE online simulation	.170	1.337	.185	.007	.476	.042	0.328	.744	.095	.202
QCAE emotion contagion	−.138	−0.995	.323	−.225	.022*	.340	2.525	.014*	.281	.006*
QCAE proximal responsivity	−.212	−1.361	.178	−.263	.009*	−.128	−0.815	.418	.100	.189
QCAE peripheral responsivity	−.036	−0.308	.759	−.098	.194	.034	0.293	.771	.073	.259

QCAE: Questionnaire of Cognitive and Affective Empathy.

* Denotes significant at *p* < .05.

As in Experiment 2, the non-biological empathic interference effect was not a significant predictor of the biological empathic interference effect, with a Bayesian simple correlation revealing moderate evidence in favour of the null (BF_01_ = 6.86). Furthermore, once the non-biological interference effect was controlled for, none of the QCAE subscales were significant predictors of the biological empathic interference effect. However, in this sample, the QCAE emotion contagion subscale was a significant predictor of the non-biological empathic interference effect.

See Figure 2 for individual participant level biological and non-biological interference effects. To ensure that the lack of a relationship between the biological and non-biological empathic interference effects was not related to low statistical power, we repeated the regression analyses using the combined data from both Experiments 2 and 3. In this larger sample, there was still no relationship between the biological and non-biological empathic interference effects (simple correlation: *r*_162_ = .045, *p* = .571). The QCAE emotion contagion subscale was significantly correlated with the non-biological empathic interference effect (*r*_162_ = .173, *p* = .027) and remained a significant predictor of this effect (β = .247, *t* = 2.654, *p* = .009) once the biological empathic interference effect was controlled for.

### Discussion

Experiment 3 replicated the main findings of Experiment 2: the empathic interference effect was of equal magnitude in the biological and the non-biological conditions, but the magnitude of the empathic interference effect for biological stimuli was not predicted by that for non-biological stimuli. This result supports the suggestion that the biological and non-biological interference effects may be regulated by two different cognitive processes.

The associations between the empathic interference effects and self-reported empathy were inconsistent across Experiments 2 and 3, however: once each of the alternative interference effects were controlled for, the only association that remained significant was that between the emotion contagion subscale of the QCAE and the non-biological empathic interference effect. In this sample, higher self-reported emotion contagion predicted a larger non-biological empathic interference effect.

## General discussion

Humans are thought to “mirror” others’ emotional responses: evidence from brain imaging shows neural overlap between observed and experienced emotion (Coll et al., 2017; Jackson et al., 2005; Singer et al., 2004). Behavioural studies also demonstrate a reaction time cost, when judging body part laterality, of observing others’ pain: the so-called empathic interference effect (Gu et al., 2010, 2012). However, if the tendency to mirror others’ emotions is not regulated, it may lead to personal distress—where individuals experience others’ negative emotions as their own (Decety & Lamm, 2009). The current study, via a modified version of the pain observation task and response times for laterality judgements (Gu et al., 2010, 2012), sought to determine whether the regulation of empathic interference is specific to biological stimuli or instead is the result of domain-general cognitive processes such as attention to salient stimuli, or cognitive control.

Experiment 1 validated a new set of stimuli which included both biological and non-biological stimuli in both painful and non-painful states. The biological pain stimuli were rated as being more painful than the other three stimulus types, validating their use as pain-evoking images.

Experiments 2 and 3 replicated the previously observed empathic interference effect whereby painful states delivered to biological stimuli lead to longer response times when making a laterality judgement about those stimuli (Brewer et al., 2018; Gu et al., 2010, 2012). However, these experiments also demonstrated that an effect of similar magnitude is observed for non-biological stimuli. This finding makes it plausible that the empathic interference effect might not arise from mirroring of the other’s painful state and subsequent regulation of such mirroring, but instead may be caused by other properties of the stimulus, such as attention to the contact between a pain-producing object and another object (whether biological or non-biological), or the fact that the pain-producing object obscures the information needed to perform a laterality judgement. Alternatively, the empathic interference effect might not be generated by emotional mirroring and its regulation for biological stimuli, but by some other domain-general cognitive process for non-biological stimuli. Under this alternative account, the mechanism by which one regulates emotional mirroring would still be considered socially specific.

In order to distinguish between these possibilities, we tested whether the magnitude of the non-biological interference effect predicted that of the biological empathic interference effect, and furthermore whether self-reported empathy predicted either effect when controlling for the other. The non-biological interference effect did not predict the biological interference effect, suggesting that the regulation of the two effects may be governed by separate cognitive processes. However, it is worth noting that, whilst every effort was made to precisely match stimuli between conditions, a lack of association between non-biological and biological interference effects cannot necessarily be considered conclusive evidence if factors other than the animacy of the stimuli play into our measurement here. Moreover, an alternative theory worth clarifying in future investigations, is that the non-biological objects may elicit biological empathic interference effects in some individuals due to them cognitively “filling” the gloves and boots with hands and feet based on prior experience. Thus, an interesting avenue for future task adaptation would be to include non-biological objects in which one would not expect to see a hand or foot such as a mug or vase.

In addition, the associations with self-reported empathy differed for the two effects. Experiment 2 indicated that greater self-reported emotion contagion and empathic concern were both associated with a larger biological empathic interference effect, and continued to predict this effect once the non-biological interference effect was controlled for. In contrast, the only (negative) predictor of the non-biological interference effect was self-reported peripheral responsivity. However, these associations were not replicated in Experiment 3. Although Experiment 3 also found an association between self-reported emotion contagion and the biological interference effect, this was in the opposite direction to that in Experiment 2, and did not remain once the non-biological interference effect was controlled for. Instead, Experiment 3 indicated that self-reported emotion contagion predicted a greater non-biological interference effect.

Despite the inconsistencies between Experiments 2 and 3 regarding the precise associations with self-reported empathy, the lack of an association between the biological and non-biological empathic interference effects, along with the finding that the two interference effects showed different patterns of associations with self-reported empathy, indicates that the two effects may be generated and/or regulated by two separate cognitive processes. Importantly therefore, and in particular as the effects are of similar magnitude, the present data highlight the need to control for the processes involved in producing the non-biological interference effect, by including and controlling for a non-biological version of the task, when using the empathic interference task in future studies.

The inconsistent relationships between the two interference effects and self-report measures of empathy have two prominent implications. The first relates to the potential validity of the task as a measure of empathic processing. The current task arguably is not fully representative of everyday scenarios in which empathy occurs: for example, it omits details about the individual to whom, and context in which, an empathic response is evoked. This is unlike other tasks of empathic processing (e.g., The Multifaceted Empathy Test: Dziobek et al., 2008; The Empathic-Accuracy Task: Zaki et al., 2008). However, the empathic interference effect is an attractive option for measuring empathic processing in part because it does not rely on self-report to measure empathic ability. Self-report may be inaccurate for at least two reasons. First, it relies on participants being able to accurately assess their own levels of empathy, an ability at which humans appear to be substantially flawed (Dunning et al., 2003; Epley, 2008; Nisbett & Wilson, 1977). Traits such as narcissism and even positive affect have been reported to impact on such metacognitive processes (Ames & Kammrath, 2004; Bower, 1991; Devlin et al., 2014). Second, the requirement to accurately and honestly report those levels, potentially introduces social desirability biases (Paulhus & Reid, 1991; Van de Mortel, 2008). It is therefore perhaps not entirely surprising (nor necessarily concerning) that the biological empathic interference effect does not appear to have a consistent relationship with self-report measures of empathy. However, future work should attempt to validate this task further, against a wider range of empathy measures.

The second implication relates to a wider issue in the social cognition literature, regarding the use of cognitive tasks to take an individual differences approach to social cognition. As recently highlighted by Qureshi and colleagues (2020), cognitive tasks typically contain features (e.g., multiple trials per cell of the design) designed to reduce the impact of individual differences on task performance. This may mean it is harder to find reliable associations between such tasks and traditional individual difference measures which are designed to optimise the measurement of individual differences. Future work could, however, provide further data on the measurement characteristics of the present task, for example, through assessment of test–retest reliability.

A final important point requiring consideration is the relationship between these experimental interference effects and the cognitive processes involved in everyday empathy. More specifically, it might be that increased interference effects of pain observation (or: increased empathic interference effects) are indicative of less successful *regulation* of emotional representations, as is the case in other domains where self-other interference is observed (e.g., imitation and theory of mind). However, due to the lack of an objective measure of participants’ emotional states in the current study, apart from the subjective ratings given in Experiment 1, it is not possible to rule out that smaller interference effects may arise via different means, such as an overall lack of emotional mirroring. Future investigations may address this possibility by combining the current task with a subjective measure of emotional arousal such as skin conductance.

In conclusion, the present paper reports the development of a new version of the pain observation task, which measures the empathic interference effect for both biological and non-biological stimuli. This builds on an ongoing body of research regarding the specificity of the mechanism required to regulate mirroring of others’ states, whether this concerns actions, mental states, or emotions. The interference effects were robust and of similar magnitudes across a discovery and replication sample. The present data suggest that different cognitive processes underlie the biological and non-biological empathic interference effects, and thus the human ability to regulate emotional mirroring may be at least in part socially specific. The current findings also highlight the importance of controlling for domain-general cognitive processes when measuring empathic interference using this task in future.
